# Effect of Test History at Ages 50–64 on Later Cervical Cancer Risk: A Population-based Case–control Study

**DOI:** 10.1158/2767-9764.CRC-23-0191

**Published:** 2023-09-11

**Authors:** Henric Kultalahti, Sirpa Heinävaara, Tytti Sarkeala, Maiju Pankakoski

**Affiliations:** 1 Finnish Cancer Registry, Helsinki, Finland.; 2 Faculty of Medicine, University of Helsinki, Helsinki, Finland.

## Abstract

As life expectancy increases, the effectiveness of cervical cancer screening programs needs to be reassessed for the older population. We addressed the effect of test history in and outside organized screening at age 50–64 years on later cervical cancer risk. A case–control study was conducted by deriving 229 cases of 65–79 years old women with invasive cervical cancer in 2010–2019 from the Finnish Cancer Registry. Ten controls were matched for each case by birth year and hospital district. The effect of test uptake and abnormal results in 50–64 year olds on cancer risk was investigated using conditional logistic regression and adjusted for self-selection. Test uptake within the 50–64 years age group showed 75% lower odds of cervical cancer [adjusted OR (aOR) = 0.25; 95% confidence interval (95% CI), 0.18–0.35]. Untested women had 4.9 times higher odds than those tested with normal results (aOR = 4.86; 95% CI, 3.42–6.92). Having at least one abnormal test result increased the odds by 2.5 when compared with only normal results but showed lower odds when compared with untested women. The importance of testing is exhibited by the result showing a reduction of odds of cancer to one-fourth for those tested compared with untested. Similarly, receiving abnormal results was protective of cancer compared with having no tests highlighting the importance of proper follow-up. Therefore, screening history should be considered when further developing cervical cancer screening programs with special interest in non-attenders and those receiving abnormal results at older ages.

**Significance:**

To our knowledge, this is the first study from Finnish data describing the effect of test history on later cervical cancer at older ages. Focusing on the cervical tests taken within the Finnish national screening program and outside it highlights the overall importance of having cervical tests and adds this study into the slowly increasing number of studies considering all cervical testing in Finland.

## Introduction

There are no uniform guidelines for when to cease cervical cancer screening programs, with some countries screening under 64 year olds and others even to 70 and over (1). Indeed, studies have suggested that screening women ages 65 or even older is effective in preventing later invasive cervical cancer (2, 3). Many countries acknowledge the need to extend screening as life expectancy increases, and as mortality in women ages 65+ is higher due to the higher probability of advanced-stage disease (4–6). However, the cervical test history in older ages also has an effect on later cancer risk.

Research suggests that having only normal test results at the age of 50 or older is associated with lower risk of developing cervical cancer in older age compared with those having received one or more abnormal results (7–10). In addition, attending testing has shown significant risk reductions especially if results were normal (11–14). For exploration of cervical cancer screening's efficacy in older ages, it is therefore important to consider history of attendance, tests outside the program, and results.

We conducted a population-based case–control study to address the effect of test history on later invasive cervical cancer risk in older women. Test history included tests taken both in and outside the organized screening program. We aimed to provide insight for policymakers on whether the screening program's cessation should depend upon recent test uptake and possible abnormal results.

## Materials and Methods

### The Finnish National Screening Program

Within the course of our study period in 2000–2019, the Finnish national screening program invited women ages 30–60 to cervical cancer screening. Municipalities were obliged to organize the screening, and some municipalities invited also 25 and/or 65 year olds. During the study period, conventional cytology was mostly used, both in and outside the screening program. Approximately 4% of the program tests in women ages 50–64 were human papilloma virus (HPV) tests with a cytology triage, performed within a randomized trial in 2003–2012 (15), or in a few municipalities that implemented HPV screening since 2012 (16).

The invitations were sent every 5 years, and in cases of milder abnormalities, such as atypical squamous cells of undetermined significance (ASC-US) or testing positive for HPV+, follow-up invitations were sent after 1–2 years. Tests with more severe abnormalities (low-grade squamous intraepithelial lesion [LSIL]+ or HPV+/ASC-US in follow-up) led to a referral to colposcopy and biopsy. All data regarding the national screening program invites, visits, and results are registered at the Mass Screening Registry. Tests outside the program are not gathered by the organization. The management of the tests outside the program follow the same guidelines as stated for the national screening program (17) but lack similar quality control as they are not recorded into a centralized registry.

### Cases and Controls

The cases in our study were women diagnosed with invasive cervical cancer (ICD-O-3 topography codes C53; ref. 18) between 2010 and 2019. Retrieved from the Finnish Cancer Registry, we identified cases who turned 65–79 years of age at the year of the diagnosis (index date). For each case, 10 controls were matched on birth year and area of residence (hospital district) at the index date. The controls were obtained from the Population Information system and were randomly selected from among women who were alive, free of invasive cervical cancer, permanent residents of Finland, and had no record of hysterectomy by the index date. For the latter, hysterectomy records (partial or total) in 1990–2019 were linked from the Care Register for Health Care (HILMO). In addition, data on education at the index date were obtained from Statistics Finland.

### Data on Test Uptake and Test Results

We derived data on tests within the Finnish national screening program between 2000 and 2018 from the Mass Screening Registry. The screening invitations covered 95%–100% of the target population during the whole study period (16). Data on tests outside the program were gathered from pathology laboratories, the nationwide Health Insurance Reimbursement Register (Kela), and the Finnish Student Health Services (19). All major laboratories operating at the time of the study delivered us data, except for a few small laboratories in the northwest part of the country. We assume a nearly complete data on tests taken between 2000 and 2014 with some tests recorded until 2017 (20).

The Bethesda system for reporting cytology (21) was mostly used in and outside the screening program, enabling harmonization of the test results between different sources. All sources were combined by using the unique personal identification code, and any duplicates were removed on the basis of the date of the test.

Test uptake was investigated by dividing tests into those taken within the Finnish national screening program and those taken outside the program. For those tested, the most severe test results at age 50–64 were categorized as follows: normal, abnormal (any abnormal cytology or HPV positive), and unknown. The categorization of test results and their hierarchy in the analysis are further explained in Table 1.

**TABLE 1:** The hierarchical order used in the definition of the most severe test result variable at age 50–64. For example, if a person was diagnosed with an HPV-positive result at least once at age 50–64, the test result was categorized as “Abnormal”, despite other possible results during that time

Hierarchy	Test result	Explanation
1	Abnormal	ASC-US, HPV positivity, referral to colposcopy, precancerous lesions
2	Normal	Normal cytology or HPV negativity
3	Unknown^a^	Test result was not clarified
4	No test	Person did not attend cervical testing

^a^Unknown cervical test results were mostly from the Health Insurance Reimbursement Register (Kela).

Evaluation of the preventive effect of testing was ensured by excluding all cervical tests taken 12 months before the index date. In other words, we neglected tests that were likely associated with the period leading to cancer diagnosis.

### Statistical Analysis

**FIGURE 1:**
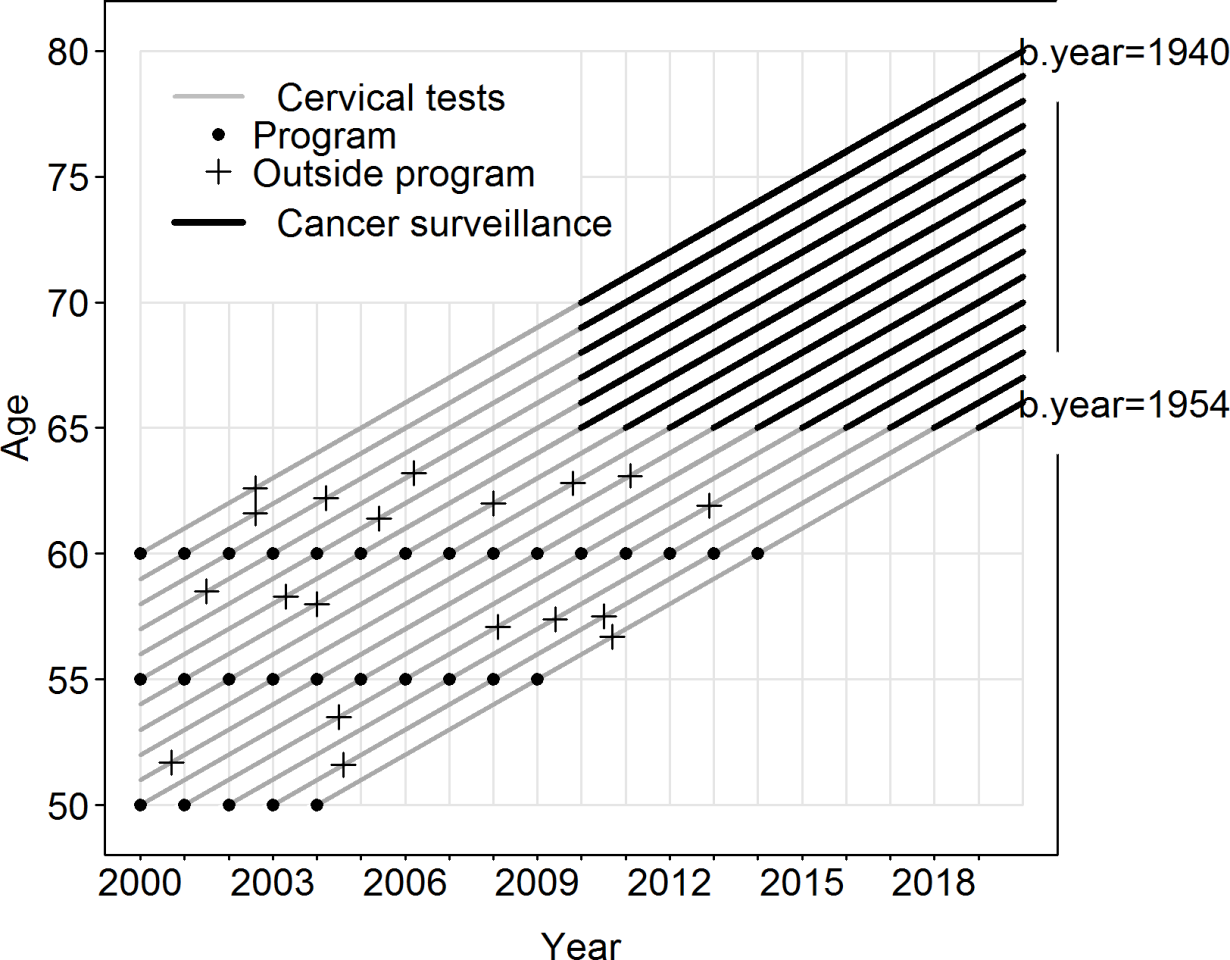
Lexis chart describing the data. The study period was 2000–2019, and the subjects were born between 1940 and 1954. The diagram shows the years when each cohort was invited to screening. Those born between 1950 and 1954 were invited to screening three times but had the shortest period of surveillance data. Those born between 1940–1944 had only one invitation to the screening program but had extensive cancer surveillance data. The figure is not indicative of the real data, but merely illustrates when testing in and outside the program can occur.

Being tested at least once within or outside the screening program and having possible abnormal results at 50–64 years were used as an exposure. The exposure to screening in our study setting depended upon the birth cohort: those born in the early 1940s were invited to screening only once but had a longer cancer surveillance period than those born in the early 1950s who were invited three times. The distribution of cancer surveillance data between these birth cohorts is illustrated in Fig. 1.

We investigated the effect of test uptake and the most severe test result at ages 50–64 on invasive cervical cancer diagnosis at ages 65–79 using conditional logistic regression. The models were adjusted for education, municipality type, and for test uptake after exiting the screening program. Following the definition by Statistics Finland, municipality type was categorized into urban, semi-urban, and rural, and education into primary, secondary, and tertiary. Educational level was used as an estimate of socioeconomic status.

Data on tests outside the program were extensively available until year 2014 and only to a limited degree after that. Thus, sensitivity analyses were performed by restricting the study population to index years of 2010–2015 to investigate possible bias from missing outside program test data. Statistical analyses in our study were performed using the R program version 4.1.1 (22).

### Data Availability Statement

The data generated in this study are not publicly available due to availability compromising patient confidentiality. Data are available from the Finnish Cancer Registry for researchers who meet the criteria for access to confidential data (i.e., research permit via Findata).

### Ethical Approval

This study was approved by the Finnish Institute for Health and Welfare (permit no. THL/1159/14.06.00/2022) and was conducted in accordance with the Declaration of Helsinki. Written informed consent is not required for register-based studies in Finland.

## Results

The study population consisted of 2,517 subjects, as a total of 229 invasive cervical cancer cases were identified and 2,288 controls were matched. Most of the cancers were diagnosed at ages 65–69 (55.5%), right after the national screening program ceased (Table 2). Out of the 229 cancers, 48% were squamous cell carcinomas and 39% were adenocarcinomas, with the rest being other types or unspecified. Most women had information on test uptake between ages 55–64. Only about 19% belonged to a cohort that could partake in all three invitational rounds within the organized screening program. Controls were more likely to have had secondary or tertiary education than cases, and testing above the screening age was also more common among controls. In addition, a slightly larger number of controls were residents of urban cities compared with cases (67.2% and 63.8%, respectively; Table 2).

**TABLE 2:** Characteristics of data

	Cases (%)	Controls (%)
	229 (100)	2,288 (100)
Age at index year		
65–69	127 (55.5)	1,270 (55.5)
70–74	77 (33.6)	768 (33.6)
75–79	25 (10.9)	250 (10.9)
Birth cohort (number of program rounds)		
1940–44 (1)	86 (37.6)	860 (37.6)
1945–49 (2)	99 (43.2)	988 (43.2)
1950–54 (3)	44 (19.2)	440 (19.2)
Educational level		
Primary or unknown	115 (50.2)	891 (38.9)
Secondary	67 (29.3)	765 (33.4)
Tertiary	47 (20.5)	632 (27.6)
Tests taken at 65+ years		
No	166 (72.5)	1,511 (66)
Yes	63 (27.5)	777 (34)
Municipality type		
Urban	146 (63.8)	1,538 (67.2)
Semi-urban	46 (20.1)	379 (16.6)
Rural	37 (16.2)	371 (16.2)

Out of the study subjects, 56% were tested outside the program at ages 50–64. Any cervical testing at ages 50–64, that is, having at least one recorded cervical test during the age range, significantly reduced the odds of cervical cancer compared with no testing [adjusted OR (aOR) = 0.25; 95% confidence interval (95% CI), 0.18–0.35] (Table 3). Those having tests both within and outside the screening program had 78% lower odds of cervical cancer compared with non-tested (aOR = 0.22; 95% CI, 0.15–0.32) but the difference between the modes of testing was quite small and the estimates had overlapping CIs (Table 3). Having cervical tests only within the national screening program reduced the odds of cervical cancer by 74% (aOR = 0.26; 95% CI, 0.18–0.39) whereas having tests only outside the program showed a 67% reduction (aOR = 0.33; 95% CI, 0.19–0.55).

**TABLE 3:** Test uptake for cases and controls at ages 50–64 in and outside the screening program. aOR for developing invasive cervical cancer at ages 65–79 compared with those not tested

Test mode	Cases (%)	Controls (%)	aOR^c^	95% CI
Not tested	74 (32)	258 (11)	1	Reference
Tested – any test	155 (68)	2,030 (89)	0.25	0.18–0.35
Tested – only program^a^	60 (26)	721 (32)	0.26	0.18–0.39
Tested – only outside^b^	23 (10)	263 (11)	0.33	0.19–0.55
Tested – both	72 (31)	1,046 (46)	0.22	0.15–0.32

^a^Only program: individuals who only had cervical tests within the national screening program.

^b^Only outside: individuals who only had cervical tests outside the national screening program.

^c^OR adjusted for education, municipality type, and having tests at age 65+.

The different test results and their ORs at age 50–64 compared with both no testing and normal results are depicted in Table 4. Those who had had cervical tests and received only normal results had an almost 80% lower odds of cervical cancer compared with those who had not been tested (aOR = 0.21; 95% CI, 0.14–0.29). Similarly, those who had received an abnormal result had an almost 50% lower odds compared with those without tests (aOR = 0.53; 95% CI, 0.34–0.83). They, however, had 2.6 times higher odds of developing cervical cancer than those having received only normal test results (aOR = 2.57; 95% CI, 1.74–3.80).

**TABLE 4:** Test results for cases and controls at ages 50–64. aOR for developing invasive cervical cancer at ages 65–79 compared with those not tested and to those with normal results

			Compared with “no tests”		Compared with “normal”	
Most severe test result	Cases (%)	Controls (%)	aOR^b^	95% CI	aOR^b^	95% CI
*Not tested*	74 (32.3)	258 (11.3)	1	Reference	4.86	3.42–6.92
*Tested – normal*	104 (45.4)	1,661 (72.6)	0.21	0.14–0.29	1	Reference
*Tested – unknown*	10 (4.4)	114 (5)	0.35	0.17–0.71	1.68	0.83–3.39
*Tested – abnormal* ^a^	41 (17.9)	255 (11.1)	0.53	0.34–0.83	2.57	1.74–3.80

^a^The abnormal category consists of results with ASC-US/HPV+, referral to colposcopy, and precancerous lesions.

^b^OR adjusted for education, municipality type, and having tests at the age of 65+.

Adjusted sensitivity analyses showed little to no difference in aORs compared with unrestricted analyses. Largest differences were seen in the test mode analyses of only outside program tests (aOR = 0.30; 95% CI, 0.15–0.61), and tests both within and outside the program (aOR = 0.17; 95% CI = 0.10–0.30; Supplementary Table S1). Otherwise, the results were similar to the unrestricted analyses (Supplementary Tables S1 and S2).

## Discussion

### Main Findings and Interpretations

This case–control study addressed the effectiveness of cervical testing in the older population in Finland. Overall, having cervical tests at ages 50–64 (during the last three rounds of the screening program) effectively prevented invasive cervical cancer in older ages. There was no significant difference between the tests taken within or outside the screening program highlighting the mere importance of having been tested.

Even those who received an abnormal result had lower odds of later invasive cervical cancer compared with those having had no tests. However, abnormal results increased the odds of cancer compared with those with normal results. These findings indicate the importance of proper follow-up of abnormal results after the termination of the screening program.

### Strengths and Limitations

In Finland, this is the first time that the effect of test uptake and results on cervical cancer risk in 65 year olds and older has been studied. We were able to minimize selection bias in controls by setting specific matching criteria and then randomly selecting them from the Population Information system database. As we chose population-based case–control setting for our study, we were able to estimate ORs in a reliable manner (23). In addition, defining exposure as having tests either within or outside the program provided a straightforward estimate for the importance of having been tested.

The data used in our study was register based and of high quality (24), and we were able to exclude hysterectomized individuals. Furthermore, we had comprehensive data on tests outside the screening program, which has only recently been available for research (20). However, like all studies, the current one had some limitations. Unfortunately, we were not able to analyze the testing age in more detail because only a minority of the women had information on all three screening rounds due to the length of the follow-up period. Information on cervical testing was mostly from one or two of the latest screening rounds, and therefore, the exposure may be interpreted as testing at late middle age.

The data on tests outside the program were mostly gathered from 2000 to 2014. To investigate the bias caused by the possibility of opportunistic tests in 2015–2018, we performed sensitivity analyses by restricting the data to index years 2010–2015 which somewhat narrowed the amount of study subjects. The results showed only a slight decrease in aORs, mostly in the test mode analyses, which could be anticipated. In addition, some part of the data on tests outside the program had no information on test results at ages 50–64. However, only approximately 5% of the women had only unknown test results, and they were treated as a separate group in the analysis to avoid possible bias. Unfortunately, we were not able to define whether the tests outside the program were taken for screening purposes or based on symptoms or follow-up. However, because it was very common for the women to have tests outside the program, we are safe to assume that most of them were taken from asymptomatic women with a purpose to screen. In addition, to effectively rule out diagnostic testing, we excluded any tests taken 12 months before the index date.

Alongside adjusting for test uptake in those ages 65 and older, we opted to minimize selection bias also by adjusting our models for municipality type and education, the latter being the best estimate for socioeconomic status in a group of 65–79 year old pensioners. However, education does not fully capture the socioeconomic status of these women, as socioeconomic status in older generation could be largely dependent on a spouse's education and income. We were not able to differentiate between primary education and unknown educational status, and thus they were grouped into the same category. However, those with an unknown education are not likely to have more than basic education. Combining these groups also eased comparison with other studies. Cases and controls differed slightly in their residential municipality types with controls being more likely to live in urban municipalities. Sharp and colleagues reported that for many cancers, including cancer of the cervix, the risk is higher in urban cities (25), which is why we controlled the analyses for municipality type.

### Comparison with Other Studies

The current study is in line with recent knowledge of screening effectiveness. In their cohort study, Wang and colleagues found that risk of cervical cancer in 60–65 year olds is largely dependent on screening history in their 50s, and that continued screening for those in high risk (i.e., unscreened, and those having received low- or high-grade abnormalities) reduces future cancer risk (7). Also other studies have found screening older women to be effective (3, 12), and suggested 5-year screening intervals to be as effective as shorter intervals (3, 26). We found that testing outside the screening program alongside attending the program barely affected the risk reductions, which supports the effectiveness of a 5-year screening interval. In addition, testing outside the program increases the cost of screening, rendering it a liability to cost-effectiveness.

A similarly conducted study to the current one from the United Kingdom showed negative results at ages 50–64 to be a protective factor after the exit age (12). The reduction of odds compared with unscreened, 84% (OR = 0.16; 95% CI, 0.13–0.19), was similar to our findings, 78%. An earlier study by Lönnberg and colleagues found a 51% odds reduction from screening in both 50–64 and 65–69 age groups compared with unscreened women in the same age group (14). Unlike earlier Finnish studies, we were able to include test results and tests outside the screening program. The addition of these tests aided us in determining the overall importance of cervical test uptake.

### Other Considerations

The decree of cervical cancer screening in Finland recently changed to include screening of 65 year olds (27). Indeed, many European countries already screen 65+ year olds, and the screening intervals vary from place to place (28). Notably, most deaths from cervical cancer occur above the screening age (4, 29), and new or reactivated latent HPV infections are reported to some extent in the older population (30, 31). In many developed countries, the age-specific cervical cancer incidence curve shows a second peak at around age 65 (32), which may reflect the lower number of lifetime screens in the oldest age groups (33). In Finland, screening coverage in women ages 55 and 60 remained low until the 1990s (34). Therefore, the incidence peak at older ages might level off in future as screening histories become more similar across birth cohorts. On the other hand, the hysterectomy rate has declined considerably in Finland (35), which may also have an opposite impact on cancer incidence at older ages (36) as it increases the number of women at risk for developing cancer of the cervix. All in all, as cervical cancer continues to affect the older population for decades to come, optimal strategies for the screening of older women are needed. Further development of screening strategy could depend upon history of screening participation and test results (13, 37), and possibly even history of previous cancerous lesions (38, 39). Modeling cost-effectiveness of these possible changes in the program would be of major interest in the future.

The high-risk HPVs are the main agents causing cancer of the cervix (40), which is why many countries have implemented HPV screening. Research suggests that the protective effects of HPV screening are longer than conventional cytology (41, 42) and could therefore potentially be effective for screening in the older age groups. For instance, a recent study by Andersen and colleagues found that HPV screening almost doubled the sensitivity but somewhat lowered the specificity in detection of severe cervical lesions compared with cytology (43). In addition, the risk of acquiring a new infection at these ages is lower than in younger age groups (44, 45) which is partly why some countries, including Denmark and Australia, have utilized HPV tests as exit tests (46, 47). After a negative exit test, the screening program can cease for the individual as the protection is thought to be sufficient.

From a clinical point of view, screening older women has its own difficulties when compared with younger women. For example, cytology screening presents issues with sensitivity as samples from older women have more morphologic differences leading to unnecessary colposcopies and over diagnosis (48). In addition, due to epithelial atrophy of the cervix and hormonal changes with aging, decreased or near impossible visibility of lesions in the cervix is a common problem with colposcopies among older women (48, 49). Improving clinical strategies for both screening and follow-up for the older population remains of interest and needs more attention to be implemented into cervical screening programs.

One of the key aspects in an effective screening program is an adequate participation rate. The lack of participation in screening has been repeatedly linked to lower education, non-native mother tongue, and living alone (50–53). Conversely, women of higher socioeconomic status, such as those with high education and wealth, participate to screening excessively by using opportunistic testing frequently (20), and the same phenomenon is evident in the older population as well (54). Indeed, opportunistic testing may exclude those with lower socioeconomic position (55, 56), as the screening program is free of charge in Finland and opportunistic testing is often paid independently (54). These individuals have a higher cancer burden due to being more likely non-attenders and having possible risk factors (57, 58), highlighting the importance of encouraging attendance to organized screening. Although our results indicate that any mode of testing is beneficial in terms of cervical cancer risk reduction, reaching maximal efficacy for the screening program should proceed by discouraging the use of opportunistic cervical tests. Frequent opportunistic testing is unnecessary for screening aged women because the screening program has been proven to be effective (14, 19), with proper quality control on each step of the screening process. These resources should be directed to improving attendance among previously non-attending older women and other high-risk groups.

### Conclusion and Policy Implications

Our main conclusion is that attendance in the national screening program in the latest rounds or having opportunistic tests taken during that time is crucial in preventing invasive cervical cancer in later years. Those who have received abnormal results should have timely follow-up in terms of screening tests.

Our results should be viewed as an implication for advancing cervical cancer screening programs to try to improve attendance especially in the older population. Screening should also be continued for middle aged women who received one or more abnormal results before exiting the screening program. In other words, the results suggest that the cessation of screening should be dependent on previous test uptake and the history of results from recent screening rounds rather than a set upper age.

## Supplementary Material

Table S1 Table S1 shows test uptake for cases and controls at ages 50–64 in and outside the screening program with data restricted to years 2000–2015, i.e. sensitivity analyses for missing opportunistic test data from 2014 onwards.

Table S2 Table S2 shows test results for cases and controls at ages 50–64 with data restricted to years 2000–2015, i.e. sensitivity analyses for missing opportunistic test data from 2014 onwards.
