# Supplementary material for: Effect of Test History at Ages 50–64 on Later Cervical Cancer Risk: A Population-based Case–control Study
Source: Cancer Res Commun. 2023 Sep 11;3(9):1823–9. doi: 10.1158/2767-9764.CRC-23-0191 (PMC10494786; doi:10.1158/2767-9764.CRC-23-0191)
Supplement: Table S1 — shows test uptake for cases and controls at ages 50–64 in and outside the screening program with data restricted to years 2000–2015, i.e. sensitivity analyses for missing opportunistic test data from 2014 onwards. [file crc-23-0191-s01.docx]

**Table S1**: Test uptake for cases and controls at ages 50–64 in and outside the screening program. Adjusted odds ratios (aOR) for developing invasive cervical cancer at ages 65–79 compared with those not tested with data restricted to 2000–2015.

| Test mode | Cases (%) | Controls (%) | aOR^c^ | 95% CI |
| --- | --- | --- | --- | --- |
| No tests | 40 (36) | 124 (11) | 1 | Reference |
| Tested - Only program^a^ | 28 (25) | 340 (30) | 0.25 | 0.14-0.43 |
| Tested - Only outside^b^ | 13 (12) | 144 (13) | 0.30 | 0.15-0.61 |
| Tested - Both | 31 (28) | 511 (46) | 0.17 | 0.10-0.30 |

^a^ Only program: individuals who only had cervical tests within the national screening program

^b^ Only outside: individuals who only had cervical tests outside the national screening program

^c^ OR adjusted for education, municipality type, and having tests at age 65+
