# Supplementary material for: Effect of Test History at Ages 50–64 on Later Cervical Cancer Risk: A Population-based Case–control Study
Source: Cancer Res Commun. 2023 Sep 11;3(9):1823–9. doi: 10.1158/2767-9764.CRC-23-0191 (PMC10494786; doi:10.1158/2767-9764.CRC-23-0191)
Supplement: Table S2 — shows test results for cases and controls at ages 50–64 with data restricted to years 2000–2015, i.e. sensitivity analyses for missing opportunistic test data from 2014 onwards. [file crc-23-0191-s02.docx]

**Table S2**: Test results for cases and controls at ages 50–64. Adjusted odds ratios (aOR) for developing invasive cervical cancer at ages 65–79 compared with those not tested and to those with normal results with data restricted to 2000–2015.

|  |  |  | Compared to no tests | | | Compared to normal | |
| --- | --- | --- | --- | --- | --- | --- | --- |
| Test mode | Case | Control | | aOR^b^ | 95% CI | aOR^b^ | 95% CI |
| No tests | 40 (35.7) | 124 (11.1) | 1 | | Reference | 5.71 | 3.49-9.36 |
| Tested - Normal | 47 (42) | 821 (73.4) | 0.17 | | 0.11-0.29 | 1 | Reference |
| Tested - Unknown | 7 (6.2) | 58 (5.2) | 0.42 | | 0.17-1.03 | 2.41 | 1.00-5.81 |
| Tested - Abnormal^a^ | 18 (16.1) | 116 (10.4) | 0.45 | | 0.24-0.87 | 2.60 | 1.45-4.64 |

^a^ The abnormal category consists of results with ASC-US/HPV+, referral to colposcopy, and precancerous lesions.

^b^ OR adjusted for education, municipality type, and having tests at the age of 65+.
